# Integrated transcriptomic and proteomic analyses of two sugarcane (*Saccharum officinarum* Linn.) varieties differing in their lodging tolerance

**DOI:** 10.1186/s12870-023-04622-z

**Published:** 2023-11-29

**Authors:** Xiang Li, Yijie Li, Ailin Wei, Zeping Wang, Hairong Huang, Quyan Huang, Litao Yang, Yijing Gao, Guanghu Zhu, Qihuai Liu, Yangrui Li, Shaolong Wei, Debin Wei

**Affiliations:** 1 Guangxi Subtropical Crops Research Institute, Nanning, 530002 China; 2 Guangxi Academy of Agricultural Sciences, Nanning, 530007 China; 3 Key Laboratory of Sugarcane Biotechnology and Genetic Improvement (Guangxi), Ministry of Agriculture and Rural Afairs, Guangxi Key Laboratory of Sugarcane Genetic Improvement /Sugarcane Research InstituteGuangxi Academy of Agricultural Sciences, Nanning, 530007 China; 4 Baise Institue of Agricultural Sciences, Baise, 533612 China; 5 Biotechnology Research Institute, Guangxi Academy of Agricultural Sciences, Nanning, 530007 China; 6 Center for Applied Mathematics of Guangxi (GUET), Guilin, 541004 China

**Keywords:** Transcriptomic and proteomic analyses, Lignification, Lodging tolerance

## Abstract

**Background:**

Lodging seriously affects sugarcane stem growth and sugar accumulation, reduces sugarcane yield and sucrose content, and impedes mechanization. However, the molecular mechanisms underlying sugarcane lodging tolerance remain unclear. In this study, comprehensive transcriptomic and proteomic analyses were performed to explore the differential genetic regulatory mechanisms between upright (GT42) and lodged (GF98-296) sugarcane varieties.

**Results:**

The stain test showed that GT42 had more lignin and vascular bundles in the stem than GF98-296. The gene expression analysis revealed that the genes that were differentially expressed between the two varieties were mainly involved in the phenylpropanoid pathway at the growth stage. The protein expression analysis indicated that the proteins that were differentially expressed between the two varieties were related to the synthesis of secondary metabolites, the process of endocytosis, and the formation of aminoacyl-tRNA. Time-series analysis revealed variations in differential gene expression patterns between the two varieties, whereas significant protein expression trends in the two varieties were largely consistent, except for one profile. The expression of CYP84A, 4CL, and CAD from the key phenylpropanoid biosynthetic pathway was enhanced in GT42 at stage 2 but suppressed in GF98-296 at the growth stage. Furthermore, the expression of SDT1 in the nicotinate and nicotinamide metabolism was enhanced in GT42 cells but suppressed in GF98-296 cells at the growth stage.

**Conclusion:**

Our findings provide reference data for mining lodging tolerance-related genes that are expected to facilitate the selective breeding of sugarcane varieties with excellent lodging tolerance.

**Supplementary Information:**

The online version contains supplementary material available at 10.1186/s12870-023-04622-z.

## Background

Sugarcane (*Saccharum officinarum* Linn) belongs to the Poaceae family and is grown worldwide for its economic value in generating sugar, cane juice, paper, pulp, alcohol, xylitol, chemicals, feed, electricity, and biomass, as well as medicinal value. This entity is native to the regions of Southeast Asia and tropical South Asia [1–3]. Sugarcane contains fructose and glucose and is the cheapest energy-producing crop [4]. Sugarcane holds a position of considerable significance as one of the primary economic crops [5, 6].

The *Saccharum officinarum* species, commonly known as sugarcane, typically grows to a height of 3 to 7 m (10 to 24 feet). A significant characteristic of this plant is that over 95 percent of its biomass is located above ground. This structural feature exposes the plant to a persistent threat of lodging, a phenomenon that can drastically reduce yield and compromise the quality of the crop. The susceptibility to lodging is directly proportional to the height of the sugarcane plant, with taller specimens facing a higher risk. After lodging, this leads to problems with harvesting, reduced cane yield and reduced sugar content. In Australia, in northern Queensland, a 15–35% decrease in sugar yields has been recorded in a lodged crop compared to an unaffected crop [7, 8]. This may be due to rat damage, suckering, and stalk and stool death following lodging [9]. Furthermore, sugarcane lodging decreases the efficiency of mechanized harvesting [10–12].

Under lodging, crop stalks shift from natural vertical shapes to permanent dislocations. In the botanical realm, the phenomenon of lodging is primarily induced by natural environmental factors such as wind and precipitation. In addition, field planting operations, fertilizer concentration, planting furrows, and physicochemical properties of the soil affect lodging [13, 14]. Typically, lodging is classified as root or stem lodging [15]. Root lodging occurs when the aboveground weight of a plant exceeds the maximum weight that the root system can withstand [16]. Stem lodging occurs when the supporting force of the base does not satisfy the weight generated by the tail of the crop stem, leading to stem breakage at the base. For instance, under sudden cold onset, the tail of sugarcane is frosted and its base lacks support, resulting in fracture, which reduces the yield [17, 18]. Stem lodging is common in crops with thin stem bases, such as rice and wheat, among others [19, 20].

Domestic research on sugarcane lodging has primarily focused on improving field cultivation measures. Lodging tolerance measures of sugarcane, such as planting lodging-tolerant varieties combined with high cultivation soil, deep trench planting, and “fork prevention method” can reduce the lodging rate [21]. To cope with typhoon-induced sugarcane lodging, wind damage can be prevented by planting windbreaks, and suitable sugarcane varieties can be planted in different ecological zones, accompanied by cultivation measures with deep plowing and deep pines [22]. Breeding lodging-tolerant varieties is the most effective approach for reducing lodging. For instance, Bred Guiding 42 (GT42), obtained through conventional crossbreeding, exhibits important traits such as strong lodging tolerance, early maturity, and high yield and has now become one of the major sugarcane varieties in Guangxi [23, 24]. However, to date, there have been no relevant reports on the regulatory genetic mechanisms underlying sugarcane lodging tolerance.

Genomes are important reference information for analyzing transcriptomes and modern sugarcane cultivars GT42 and GF98-296 in this study. Contemporary sugarcane varieties are the result of hybridization efforts initiated by breeders in the early twentieth century. These efforts aimed to combine the high sucrose content of *Saccharum officinarum* with the disease resistance and stress tolerance traits inherent in *S. spontanuem*. Consequently, Saccharum cultivars now possess an extraordinarily intricate interspecific aneupolyploid genome, comprising 100 to 130 chromosomes. Approximately 70% to 80% of these chromosomes originate from *S. officinarum*, 10% to 20% from *S. spontaneum*, and around 10% result from interspecific recombination [25]. Despite the release of the whole-genome sequence of *S. spontaneum* [26] and a monoploid sequence of a commercial cultivar, R570 [27], comprehensive genomic information pertaining to modern sugarcane hybrids or *S. officinarum* remains unpublished.

Transcriptome sequencing technology has also been applied to screen sugarcane germplasm resources. Transcriptome analysis showed that sugarcane pest herbivores enhanced several herbivory-induced responses, including amino acid metabolism carbohydrate metabolism and secondary metabolites, pathogen responses, plant hormone signaling transduction, and transcription factors. This study will accelerate our understanding of the mechanisms of insect herbivory in sugarcane and provide a target for improving the resistance of sugarcane varieties to insect herbivory through molecular breeding [28]. Sugarcane transcriptome studies also are primarily used to characterize genes and their relationships with stress tolerance. Developing sugarcane varieties with high-stress tolerance is one of the main goals of researchers for increasing sugarcane yields. Therefore, understanding the sugarcane transcriptome under stress conditions is important for future crop breeding [29]. Besides, transcriptome sequencing can also be used to reveal the genetic regulatory mechanisms underlying sugarcane lodging tolerance.

Therefore, in the present study, we selected two sugarcane varieties that differed in terms of their lodging tolerance as research materials to explore the molecular mechanisms associated with lodging tolerance in sugarcane. The physiological and biochemical characteristics as well as the microstructures of the two varieties were compared. Transcriptomic and proteomic analyses were performed to identify gene regulatory mechanisms underlying lodging tolerance in sugarcane. Our results provide a basis for mining lodging tolerance genes that can aid in the selection and breeding of sugarcane varieties with excellent lodging resistance.

## Results

### Comparison of the lodging index of two sugarcane varieties

**Fig. 1:**
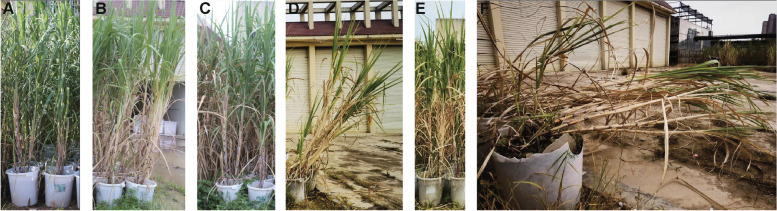
Lodging events of GT42 and GF98-296 sugarcane varieties during different developmental stages. Growth stage and lodging in (**a**, **b**) September, (**c**, **d**) October, and (**e**, **f**) November. Plants in a, c, and e represent GT42, and plants in **b**, **d**, and **f** represent GF98-296

The sugarcane growing period was from mid-September to late September. Interestingly, in Guangxi, typhoons and rain intensified in mid-to-late September; therefore, the sugarcane variety GF98-296 showed initially inclined lodging owing to defects in its cane stem structure (Fig. 1b) in comparison to the lodging-tolerant variety GT42 (Fig. 1a). In October, the height of GT42 continued to increase (Fig. 1c), whereas the angle of inclination in variety GF98-296 further increased (Fig. 1d). In November, GT42 reached its maximum height (Fig. 1e). However, the original inclined variety GF98-296 was affected by climate and its factors, and the entire sugarcane almost collapsed (Fig. 1f). Although the height data of GT42 and GF98-296 were relatively close at each period of growth (Table S1), the lodging resistance index (LRI) was obviously different between these two varieties. The LRI of GT42 (3.0) was much higher than that of GF98-296 (1.16). The ratio of middle in basal stem diameter in GT42 was also higher than GF98-296 (Detailed data are shown in Table S2).

**Fig. 2:**
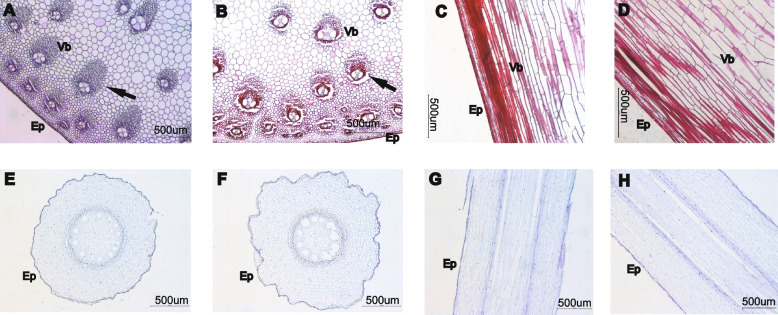
Stem microstructures of the two tested sugarcane varieties with different degrees of lodging at the mature stage. Cross section: **a**, **e** = GF98-296 and **b**, **f** = GT42. Longitudinal section: **c**, **g** = GF98-296 and **d**, **h** = GT42. Black arrow indicates Vb (EP, epidermis; Vb, vascular bundle)

Crocus (safranin O-fast green staining), was used to stain cell wall lignification and suberization. In a comparative analysis of the cross-sectional view of the epidermis, the lodging-tolerant sugarcane variety GT42 showed clear red staining of the crocus (Fig. 2), indicating a high degree of lignification and suberization. This is consistent with the lignin and hemicellulose contents in GT42 that were much higher than those in GF98-296 (Table S3). From the field view, the number of vascular bundles in the stem was higher in GT42 than in GF98-296. Moreover, ducts and fibers in the vascular bundle were obvious, and cells near the vascular bundle showed obvious tears in GT42 (indicated by arrows in Fig. 2a and b). Furthermore, the cortical thickness in GF98-296 was greater than that in GT42 (Fig. 2c and d), and the cortical cells were arranged neatly in many layers. In the field view, longitudinal sections showed more cortical cell layers in GF98-296 than in GT42 (Fig. 2g and h), with a relatively neat arrangement. In the field view, the cross section indicated more epidermal cell layers in GT42 than in GF98-296. Moreover, the native xylem duct and mid-column sheath cells were more developed in GT42. Similarly, the cortical walls and middle column parenchymal cells appeared thicker in GT42 than in GF98-296 (Fig. 2e, f). Furthermore, in the cross- and longitudinal sections, the root cortical parenchyma cells were arranged very neatly in GT42. A well-developed duct can better transport water and mineral elements to the aboveground parts, which is conducive to the growth of sugarcane plants.

### Transcriptome analysis of sugarcane stem across different stages

To identify genes related to the difference in lodging tolerance between GT42 and GF98-296, global RNA sequencing (RNA-seq) analysis of stem fragments at the three developmental stages was performed. After discarding low-quality reads, RNA-seq yielded 6,291,005,512–6,525,591,363 clean reads with Q20 bases on average per sample (Table S4), which were used for further expression analyses. The assembly of the reads produced 86,009 unigenes with an average length of 1011 bp (Table S5). The BUSCO evaluation of unigenes revealed that ~ 68% of the BUSCOs were complete, indicating relatively high completeness of the assembly (Fig. S1). All unigenes were BLASTed to multiple databases, and 45,684 unigenes were annotated to at least one database (Table S5).

**Fig. 3:**
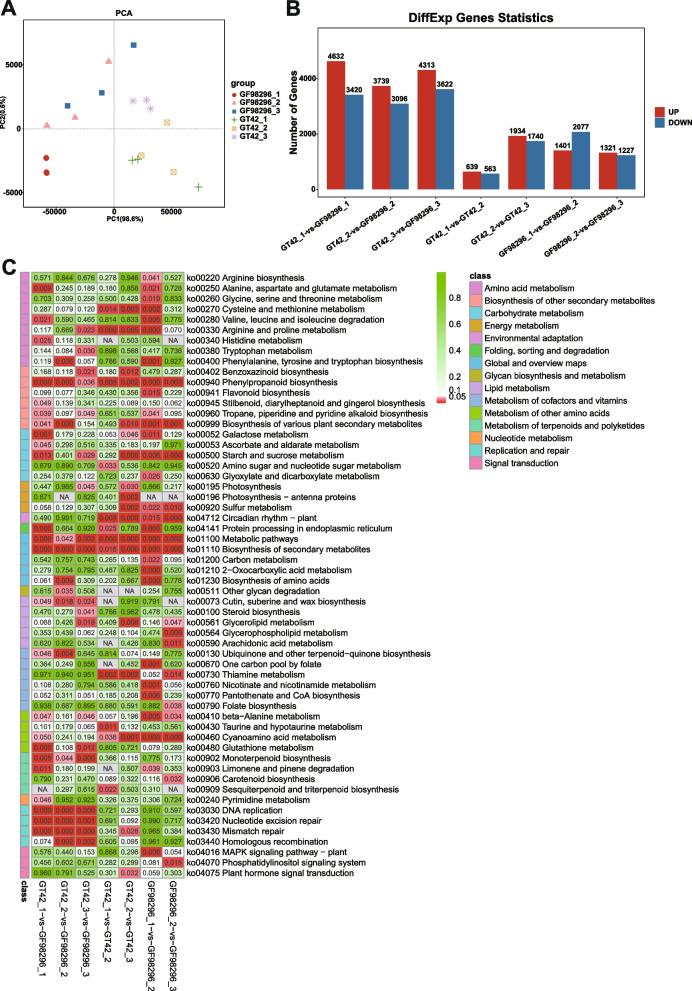
Transcriptome analysis of differentially expressed genes in sugarcane. PCA of the expressed genes (**a**); x- and y-axes indicate the first and second principal components, respectively. Bar chart of differentially expressed genes (**b**); red and blue represent upregulated and downregulated genes, respectively. KEGG pathway enrichment analysis of genes at different growth stages of GF98-296 and GT42 (**c**), was performed using the KEGG software of the Kanehisa laboratory

Principal component analysis (PCA) was conducted to revealed the relationship among different samples (Fig. 3a). Samples in the same groups tended to cluster together, indicating consistency between the samples. Samples collected from different varieties were far apart, suggesting variation between the two varieties. The differential expression analysis showed that 1,202 (639 upregulated and 563 downregulated) and 3,674 (1,934 upregulated and 1,740 downregulated) genes were differentially expressed at stages 2 and 3 compared to the previous stage in variety GT42, respectively. In variety GF98-296, 3,478 (1,401 upregulated and 2,077 downregulated) and 2,548 (1,321 upregulated and 1,227 downregulated) differentially expressed genes (DEGs) were identified at stages 2 and 3 compared to the previous stage, respectively. Besides, 8,052, 6,835, and 7,935 genes were differentially expressed between varieties GT42 and GF98-2961 at stages 1, 2, and 3, respectively (Fig. 3b).

KEGG pathway enrichment analysis was performed on the differentially expressed genes (DEGs) from each comparison. Overall, 58 KEGG pathways were significantly enriched in at least one comparison (Fig. 3c). Among these, the pathways of circadian rhythm and cyanoamino acid metabolism were enriched by the DEGs of both GF98-296 and GT42 during growth. In addition, the phenylpropanoid biosynthetic pathway, secondary metabolite biosynthetic pathways, cutin, suberin, wax biosynthesis, monoterpenoid biosynthesis, DNA replication, nucleotide excision repair, and mismatch repair were simultaneously enriched in GF98-296 versus GT42 at all growth stages.

### Proteomic changes in the sugarcane stem

**Fig. 4:**
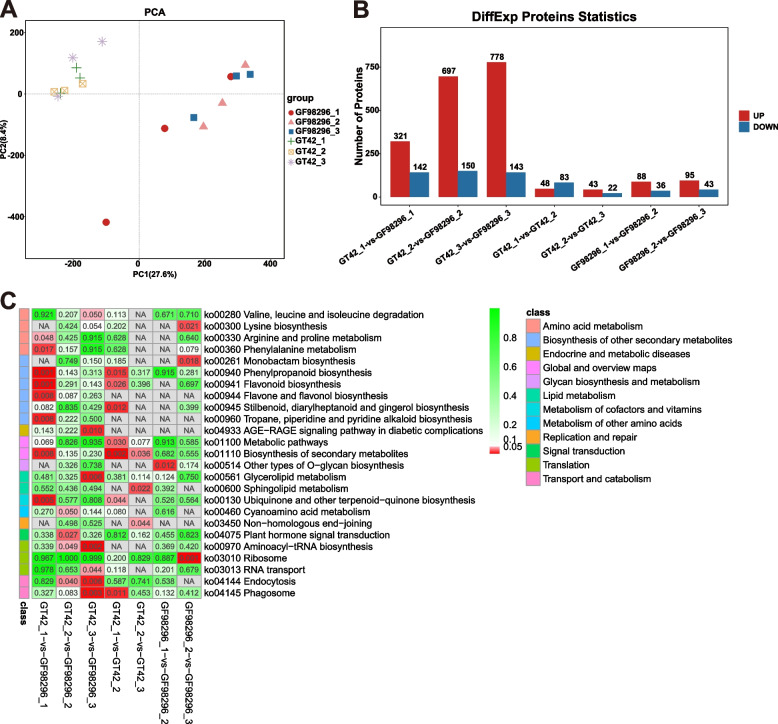
Proteomic analysis of differentially expressed proteins in sugarcane. PCA of expressed protein (**a**); x- and y-axes indicate the first and second principal components, respectively. Bar chart of differentially expressed proteins (**b**); red and blue represent upregulated and downregulated proteins, respectively. KEGG pathway enrichment analysis of proteins at different growth stages of GF98-296 and GT42 (**c**), was performed using the KEGG software of the Kanehisa laboratory

Proteomic analysis of 18 GT42 and GF98-296 samples identified and quantified 46,462 peptides and 6,551 proteins (Dataset 1). According to the PCA plotting, samples in the same group tended to cluster, indicating that they were more consistent. Conversely, the samples from different varieties were far apart, suggesting that the two varieties were distinct (Fig. 4a). In variety GT42, 131 (48 upregulated and 83 downregulated) and 65 (43 upregulated and 22 downregulated) proteins were differentially expressed at stages 2 and 3 compared to the previous stage, respectively. In variety GF98-296, 124 (88 upregulated and 36 downregulated) and 138 (95 upregulated and 43 downregulated) differentially expressed proteins (DEPs) were found at stages 2 and 3 compared to the previous stage, respectively. Moreover, 463 (321 upregulated and 142 downregulated), 847 (697 upregulated and 150 downregulated), and 921 (778 upregulated and 143 downregulated) DEPs were identified between varieties GT42 and GF98-2961 at stages 1, 2, and 3, respectively (Fig. 4b).

KEGG pathway enrichment analysis showed that in GF98-296, DEPs between stages 1 and 2 were significantly enriched in other types of O-glycan biosynthesis pathways, whereas DEPs between stages 2 and 3 were significantly enriched in the ribosome, monobactam biosynthesis, and lysine biosynthesis pathways (Fig. 4c). In GT42, the DEPs between stages 1 and 2 were significantly enriched in phagosome, stilbenoid, diarylheptanoid, gingerol, phenylpropanoid, flavonoid, ubiquinone, and other terpenoid-quinone biosynthesis, whereas the DEPs between stages 2 and 3 were significantly enriched in the biosynthesis of secondary metabolites, sphingolipid metabolism, and non-homologous end-joining. In addition, the DEPs between the two varieties at different growth stages were enriched in the phenylpropanoid biosynthesis, flavonoid biosynthesis, ubiquinone and other terpenoid-quinone biosynthesis, and secondary metabolite biosynthetic pathways (Fig. 4c).

### Differential gene and protein expression patterns between the two sugarcane varieties

**Fig. 5:**
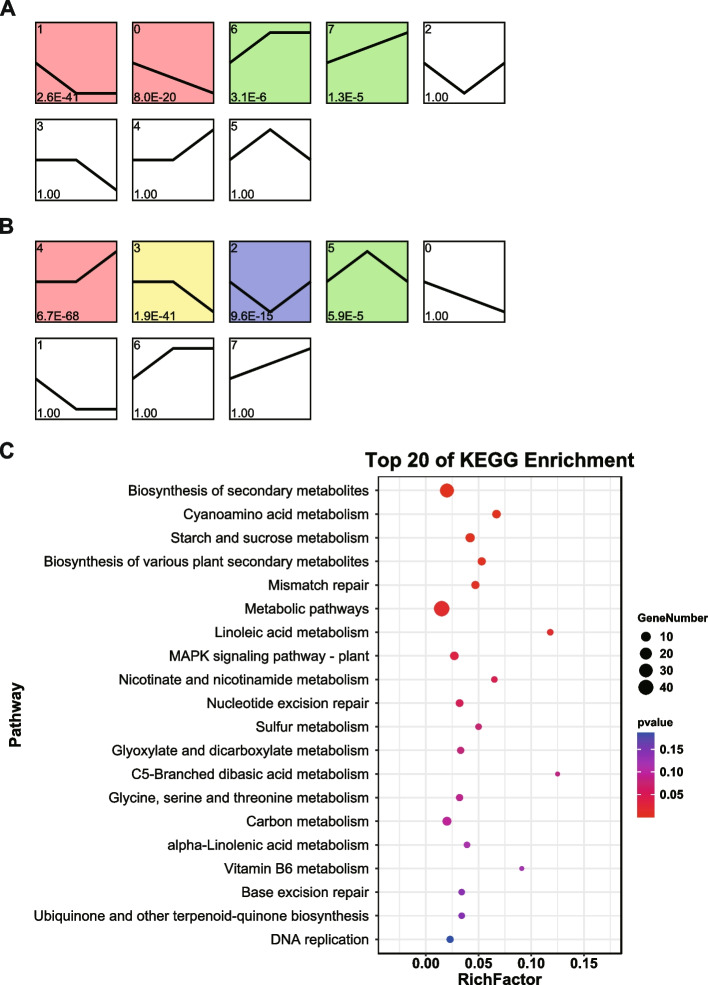
Trends of gene expression and KEGG enrichment to identify key metabolic pathways. STEM analysis of gene expression profiles of GF98-296 (**a**) and GT42 (**b**). Each box indicates a model profile, and the colored profiles shown are significant. The profiles showed by the same color can be secondary combined to simplify the trend analysis results. Numbers in the boxes indicate the order of the profile (upper left), and the P-value indicates significance (lower left). The top 20 pathways from KEGG enrichment analysis of genes (**c**). X-axis shows the enrichment factor (RichFactor), calculated as the number of differentially expressed genes annotated to the pathway divided by all genes identified in the pathway. The larger the value, the greater the proportion of differentially expressed genes annotated to the pathway. The size of the circle represents the number of differentially expressed genes annotated to the pathway

**Fig. 6:**
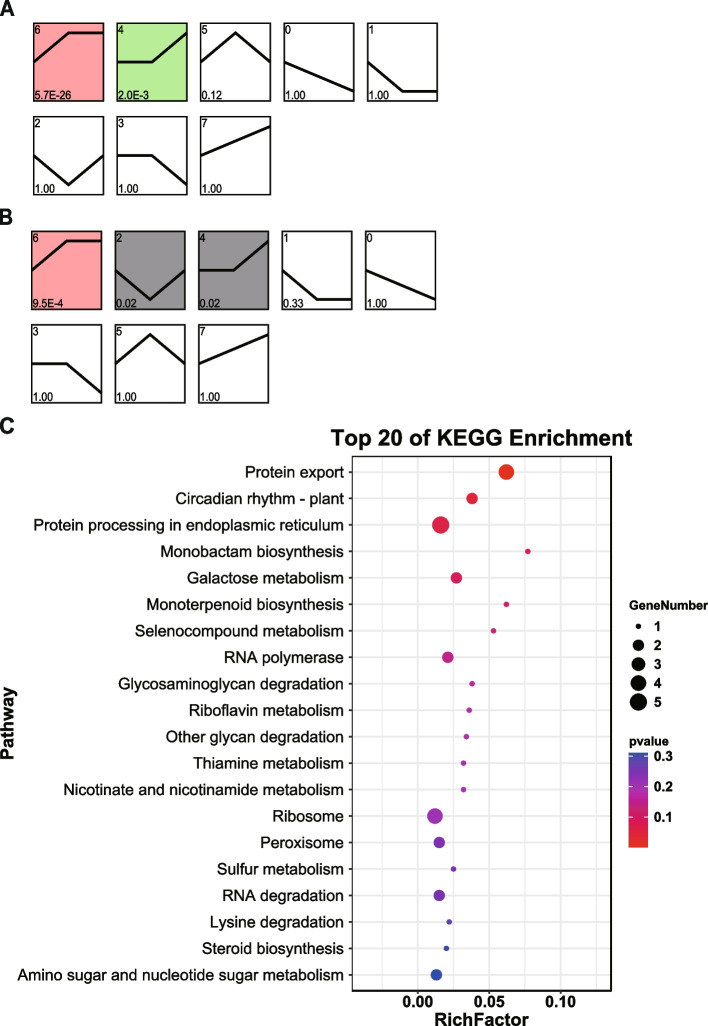
Trends of protein expression and KEGG enrichment to identify key metabolic pathways. STEM analysis of protein expression profiles of GF98-296 (**a**) and GT42 (**b**). Each box indicates a model profile. Colored profiles show significant values. Numbers in the boxes indicate the order of the profile (upper left), and the P-value indicates significance (lower left). The top 20 pathways from KEGG enrichment analysis of proteins (**c**). X-axis represents the enrichment factor (RichFactor), calculated as the number of differentially expressed proteins annotated to the pathway divided by all proteins identified in the pathway. The larger the value, the greater the proportion of differentially expressed proteins annotated to the pathway. The size of the circle represents the number of differentially expressed proteins annotated to the pathway

Gene and protein expression patterns were analyzed separately using STEM software for GF98-296 and GT42 based on the DEGs and DEPs identified from stage-dependent comparisons, which were clustered into eight profiles for each variety (Figs. 5a, b and 6a, b). The DEGs in GF98-296 were significantly enriched in profiles 1, 0, 6, and 7, whereas the DEGs in GT42 were significantly enriched in profiles 4, 3, 2, and 5 (marked by colors in Fig. 5a and b). Therefore, the trends in the DEGs differed between the two varieties. We further divided the trends into downward (profiles 0, 1, 2, and 3) and upward (profiles 4, 5, 6, and 7). Accordingly, 486 genes with differences in trend changes between varieties were identified. These genes were largely enriched in the biosynthesis and metabolism of secondary metabolites, cyanoamino acid metabolism, starch, and sucrose metabolism (Fig. 5c).

DEPs in GF98-296 were significantly enriched in profiles 4 and 6, whereas the DEPs in GT42 were significantly enriched in profiles 2, 4, and 6. The trends in DEPs between the two varieties were consistent, except for profile 2 (Fig. 6a and b). We then focused on proteins with different trends between the varieties and identified 178 DEPs. These proteins were primarily enriched in protein export, circadian rhythm, and protein processing pathways in the endoplasmic reticulum (Fig. 6c).

**Fig. 7:**
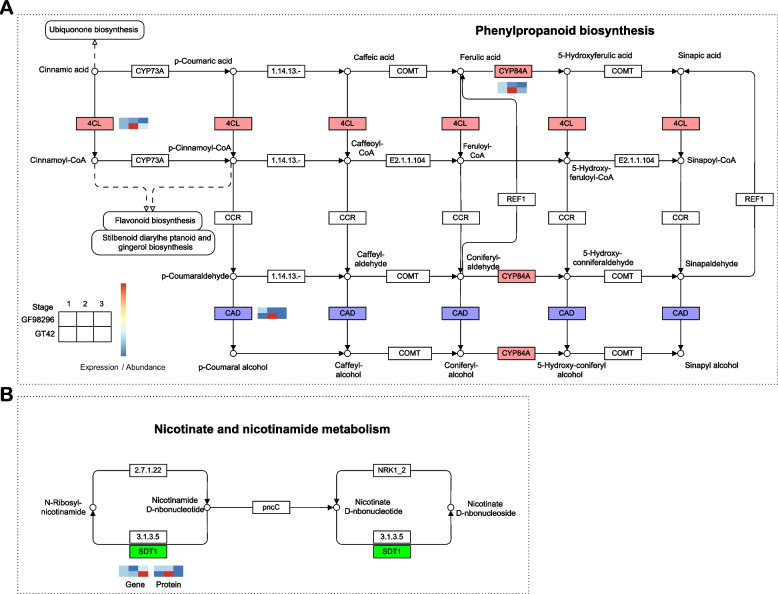
DEGs and DEPs involved in the phenylpropanoid biosynthesis (**a**) and nicotinate and nicotinamide metabolic (**b**) pathways with the permission No. 232106

To investigate the key metabolic pathways differentially regulated at the transcriptomic and proteomic levels between the two varieties, the pathways affected by both gene and protein trends were investigated (Table S6). The phenylpropanoid biosynthetic pathway harbored three key genes that were differentially expressed between the two varieties (Fig. 7a). The expression of CYP84A, 4-coumarat-CoA ligase (4CL), and alcohol dehydrogenase (CAD) was enhanced in GT42 samples at stage 2, but suppressed in GF98-296 samples at the growth stage. The key gene *sdt1* (pyrimidine and pyridine-specific 5’-nucleotidase) in nicotinate and nicotinamide metabolism was differentially expressed between the two varieties at both the transcript and protein levels (Fig. 7b). Specifically, SDT1 expression increased in GT42 samples but decreased in GF98-296 samples during the growth stage. The expression of these genes was validated using qRT-PCR (Fig. S2), which showed a high consistency between the transcriptome and qRT-PCR testing.

## Discussion

Lodging severely reduces crop yield and is therefore an important scientific research topic. Based on the lodging angle, studies on crops such as rice, maize [30], and wheat [31], have divided lodging into three or more grades. However, a standard grading system for defining the lodging angle in sugarcane has yet to be established. In this study, we propose a grading system for sugarcane lodging, which is divided into grades 1 (lodging), 2 (semi-lodging), and 3 (upright). Furthermore, we aimed to standardize the grading system by using the lodging tolerance index (LTI) of sugarcane. The LTI was first proposed for wheat, with a positive correlation between lodging tolerance and LTI value, suggesting that the larger the LTI, the stronger the lodging tolerance of wheat [32]. The proposed LTI with a grading standard can be effectively used to determine the lodging tolerance of sugarcane plants. While screening different sugarcane varieties, breeders primarily rely on visual inspection to infer lodging tolerance, which is useful if the varieties are all upright or lodged; however, drawing correct conclusions is difficult for a variety that exists in both lodging and upright forms. Assessing the lodging nature of such varieties is a challenging task because, at later stages, such varieties may tend to be completely lodged or upright, resulting in undesirable outcomes. After investigating the growth of different sugarcane varieties at different stages, the LTI obtained from our calculations can aid in estimating the lodging tolerance of sugarcane varieties, which can greatly improve the breeding efficiency of lodging-tolerant sugarcane varieties.

In vascular plants, lignin serves as a crucial structural constituent of the cell wall and is intricately linked with the processes of plant growth and development. Lignin confers physical strength to the plant body [33], thereby playing an important role in lodging tolerance. In the present study, we analyzed the microstructure of sugarcane stems and examined the association between the tissue components of sugarcane rhizomes and lodging type. Microscopic analysis of the upright (GT42) and lodged (GF98-296) sugarcane varieties revealed a high degree of lignification and suberization in the stems of the GT42 variety. Furthermore, the quantity of both large and small vascular bundles was observed to be significantly elevated in GT42 in comparison to GF98-296; the ducts and fibers in the vascular bundles were lignified; however, the epidermal cells in GF98-296 stems were larger and more neatly arranged. GT42 was enriched in the epidermal cell layers compared to GF98-296. Similarly, cortical cell walls and middle-column parenchyma cells were thicker in GT42 than in GF98-296. Moreover, an examination of the biochemical composition revealed an elevated concentration of lignin and hemicellulose in GT42 compared to GF98-296 (refer to Table S3). Conversely, the cellulose content was markedly superior in GF98-296 than in GT42. Lignin, cellulose, and hemicellulose collectively form a complex network, and any perturbation in any of these components can potentially disrupt normal function [34, 35]. Specifically, an increase in their content improves lodging tolerance [36, 37]. Furthermore, vascular bundles serve to fortify the plants. As such, the quantity of vascular bundles is a direct determinant of the mechanical strength of the stems. An augmentation in the number of vascular bundles in wheat stems can effectively bolster lodging tolerance [38], and a robustly positive correlation between the number of vascular bundles and lodging resistance has been documented in rice [39, 40]. In alignment with these observations, our analysis demonstrated a significant escalation in the number of vascular bundles in GT42 (a lodging-tolerant variety) in comparison to GF98-296.

The subsequent integration of transcriptomic and proteomic analyses elucidated disparities in the comprehensive gene expression profiles, specifically in the modulation of the phenylpropanoid biosynthetic pathway, between GT42 and GF98-296. The phenylpropanoid pathway synthesizes a variety of metabolites, including lignin, flavonoid lignans, and cinnamic acid amides [41, 42], which enhance the tolerance of stalk lodging [43]. This is a major pathway in lignin synthesis [44]. Lignin, a crucial component, contributes significantly to stem rigidity, with its concentration being notably higher in robust stems as compared to their weaker counterparts [33]. Consistently, our findings indicate that the lodging cross trait can be attributed to insufficient lignin synthesis, whereas the semi-lodging trait is premised on stable lignin content. We postulate that the cellulose and hemicellulose concentrations in GF98-296 may not attain a suitable threshold, potentially influencing the degree of lodging. This is due to the fact that cellulose serves as the primary structural constituent of the cell wall [45], and any damage to stem hardness due to insufficient lignin content can be compensated by increasing the cellulose content [46]. Three genes, which are implicated in the biosynthesis of lignin, exhibited differential expression patterns between GT42 and GF98-296 during the growth phase (Fig. 7). In particular, CYP84A, 4CL, and CAD were upregulated in GT42 samples at stage 2 but downregulated in GF98-296 samples at the growth stage, consistent with previous reports indicating that certain alterations are positively regulated toward tolerance [33, 43].

Arginine and proline metabolism was enriched in GT42 during different growth stages, specifically in GF98-296 at stage 1 and in GT42_3 vs. GF98-296_3, which may be an important pathway for drought resistance of sugarcane as it was also enriched in the metabolome of the sugarcane variety Badila under drought stress [47]. Phenylpropanoid biosynthesis was enriched in both the GT42 and GF98-296, which play important role for plants to cope with both negative and positive environmental stimuli and other forms of life [48]. Sulfur metabolism was enriched in GF98-296 during different growth stages and was specifically enriched in GT42 at stage 2, which is stimulated by photorespiration [49]. The enriched pathway was positively correlated with the lodging and upright characteristics of the sugarcane. Sugarcane undergoes four distinct stages of growth: germination, tillering, grand growth, and maturation and ripening. The elongation of sugarcane primarily transpires towards the conclusion of the grand growth phase and the initial stage of the maturation/ripening phase. Therefore, genes associated to lodging resistance might be abundantly expressed during the elongation phase (GT42 at stage 2) and their expression subsequently decreased (GT42 at stage 3) (eg. 4CL and CAD in Fig. 7a). PAL, 4CL, CAD, and COMT play vital roles in lignin biosynthesis of wheat [40, 50, 51]. In muskmelon, the upregulation of PAL, C4H, and 4CL promotes lignin biosynthesis and increases black spot disease resistance [52]. Moreover, Os4CL suppression reduces lignin content in rice [53]. These findings propose a direct association between the phenomenon of sugarcane lodging and the transcriptional activity of lignin biosynthetic genes within the phenylpropanoid pathway. These genes are valuable potential targets for improving sugarcane stalk lodging tolerance because they likely regulate lignin accumulation, which is a vital factor affecting stem strength.

The metabolism of nicotinate and nicotinamide plays a pivotal role in photosynthesis, the response of plants to stress, and the process of cellular expansion [54–56]. Specific gene families associated with nicotinate and nicotinamide metabolic pathways have been documented. For example, nicotinamide mononucleotide adenylyl transferase (NMNAT) is a crucial structural gene that governs the transformation of nicotinamide adenine dinucleotide (NAD) to NaMN [57]. Furthermore, SDT1(pyrimidine and pyridine-specific 5’-nucleotidase) is accountable for the synthesis of nicotinamide riboside (NR) in cells, and its activity is inversely proportional to cellular NAD levels [58]. These two enzymes significantly influence cellular NAD homeostasis, a routine process in NAD metabolism [57, 58]. In the present study, the expression of the key gene *sdt1* in nicotinate and nicotinamide metabolism increased in GT42 samples but decreased in GF98-296 samples during the growth stage. Therefore, an imbalance between NR and NAD may result in different lodging traits in sugarcane. Future work should focus on detecting the levels of NR and NAD to determine whether they directly affect the lodging resistance of sugarcane.

Notably, GT42_1 vs. GT42_2 and GF98-296_1 vs. GF98-296_2 showed different results for DEGs and DEPs (Figs. 3 and 4). The weak correlation between the mRNA and protein levels may result from many factors. Correlation coefficients exhibit variation across diverse organisms, with ranges from 0.2 to 0.47 in bacteria, 0.34 to 0.87 in yeast, and 0.09 to 0.46 in multicellular organisms [59]. The existence of a weak ribosome-binding site (Shine-Dalgarno for prokaryotes and Kozak for Eukaryotes), regulatory proteins, codon usage bias, and disparities in the half-life between protein and mRNA constitute some of the biological factors that could elucidate the feeble correlation observed between measured RNA and proteins [60].

In summation, we amalgamated transcriptomic and proteomic analyses to discern the disparities in gene expression profiles between upright and lodged sugarcane across various developmental stages. Specifically, the differential modulation of pathways, such as the phenylpropanoid biosynthetic pathway associated with lignin biosynthesis, and nicotinate and nicotinamide metabolism linked to NR and NAD imbalance, may be correlated with the lodging tolerance of sugarcane. These insights pave the way for future breeding endeavors aimed at cultivating sugarcane varieties with superior lodging tolerance.

## Methods

### Plant material and sampling

Two cultivars, GT42 (of the upright type) and GF98-296 (of the completely lodged type), bred by the Guangxi Academy of Agricultural Sciences (Guangxi Province, China), were employed in this investigation. The cultivars were planted according to standard management practices in a field situated at the Sugarcane Research Institute of the Guangxi Academy of Agricultural Sciences (22°51′N, 108°14′E). On March 15, 2021, the newly cut detoxified stems of GT42 and GF98-296 were placed in sand pots for cultivation, and sugarcane buds germinated on April 2, 2021. The barreled soil obtained from the test field was screened and mixed with clean river sand at a ratio of 3:1 (v/v). The specimens were procured during the months of September, October, and November. Sugarcane stems of the corresponding variety were chopped, and a sample of the stem segment was cut 5 cm from the incision on the stem to the middle of the internode in the tail direction and further divided into 1 cm fragments. Five plants were sampled from each replicate and pooled into a 15 g sample. This step was repeated thrice. The specimens were subsequently transferred into Eppendorf tubes, subjected to freezing via liquid nitrogen, and preserved at a temperature of -80 °C for future utilization.

To maintain uniformity, plants of analogous sizes at each developmental stage (grand growth phase of sugarcane in September and maturation/ripening phase of sugarcane in October and November) were chosen for transcriptomic and proteomic analyses. Three biological replicates were established for each cultivar and developmental stage to generate transcriptomic and proteomic data. After cutting the flat soil surface of the cane stem in the field root test, a stem segment with a length of 5 cm from the cane stem incision was taken from a position 5 cm away from the cane stem incision to the tail direction for analysis. Each biological replicate was procured from an aggregation of more than five plants, with a total weight exceeding 1.5 g. The samples analyzed in the present study were labeled as variety replicates, where varieties 1 and 2 corresponded to GT42 and GF98-296, and stages 1, 2, and 3 corresponded to the September, October, and November sampling stages, respectively. The total nitrogen concentration was assessed utilizing the Kjeldahl method [61], while the content of potassium and calcium were measured via a flame photometer method [62, 63]. Determination of hemicellulose, cellulose, lignin, WSP, ISP, CSP and protopectin content using FTIR in Calycophyllum spruceanum (Benth.) K. Schum. and Guazuma crinita Lam [64].

### Assessment of lodging tolerance

Lodging grade was determined according to a previously established procedure [65] with certain modifications. The lodging angle is characterized as the angle formed between the head and base of the cane stem and the vertical line of the ground. Lodging is categorized into three grades: a lodging angle greater than or equal to 60° signifies grade 1, a lodging angle ranging from 30° to 60° signifies grade 2, and a lodging angle less than or equal to 30° signifies grade 3. The LTI was calculated as described by Li et al. [66]. The LTI was calculated as follows: LTI = (number of levels × number of plants at that level) / number of investigated plants. Based on the data obtained in the present study, observations of sugarcane lodging over the years, the results of related studies, and evaluation criteria for lodging tolerance in the field were established. Specifically, 1.0 ≤ LTI ≤ 1.6 is defined as grade 1, indicating complete lodging; 1.6 ≤ LTI ≤ 2.3 is defined as grade 2, indicating tilting; and 2.3 ≤ LTI ≤ 3 is defined as grade 3, indicating the upright status. Additionally, the histochemical staining of the cell wall was conducted utilizing the Safranin O-Fast Green method, as previously delineated [67].

### RNA extraction and RNA-seq

For RNA sequencing, triplicate samples were amassed at different stages in each variety. Total RNA was isolated utilizing a TRIzol kit (Invitrogen, Carlsbad, CA, USA). After elimination of DNA contamination with DNase I (Takara Bio, Otsu, Japan), the integrity of the RNA was measured with a Bioanalyzer (Agilent Technologies, Santa Clara, CA, USA). Then the mRNA was isolated from total RNA with the Dynabeads mRNA Purification Kit (Invitrogen) and fragmented to 200–400 bp using a fragmentation buffer (Ambion, #AM8740). The cDNA library was generated from the purified mRNA following the manufacturer’s guideline of Optimal Dual-mode mRNA Library Prep Kit (BGI, Shenzhen, China) as previously described [68], and paired-end 150 bp (PE150) sequencing was perform on a BGISEQ-500 platform (BGI).

### Analysis of transcriptomic data

Raw reads underwent filtration using Fastp (v0.18.0) to yield clean reads. De novo assembly of the clean reads was performed utilizing the Trinity software package (v2.6.6) with default parameters. BUSCO (v3) was applied to evaluate the completeness of the assembled unigenes. BLAST against databases, encompassing nr protein, Swiss-Prot, KEGG, TREMBL, GO, and COG, was conducted to obtain functional annotations of the unigenes. Gene expression was quantified employing RSEM (v1.2.19). Differentially expressed genes (DEGs) between comparison groups were identified using the DESeq2 package (v1.22.2) [69], and the P-values were adjusted according to the Benjamini–Hochberg false discovery rate (FDR) method. An FDR less than 0.05 and an absolute log2(fold change) greater than or equal to 1 were applied as thresholds for filtering significant DEGs. KEGG pathway analysis of DEGs was conducted, and significant enrichment was determined using hypergeometric tests.

### Protein extraction and Data-Independent Acquisition (DIA) proteome analysis

Fusion Lumos (Thermo Fisher Scientific, San Jose, CA, USA) was used to acquire mass spectrometry (MS) data of 18 samples in DIA mode, and 46,462 peptides and 6,551 proteins were quantified. The quantification of peptides and proteins was executed utilizing the MSstats software package. The DIA analysis pipeline is based on three essential steps: spectral library construction, large-sample data acquisition in the DIA mode, and data analysis. Proteins were isolated from the stem samples of sugarcane, and the quantification of these proteins, as well as the assessment of the quality of the extraction, were performed using the Bradford quantification method and Sodium Dodecyl Sulfate–Polyacrylamide Gel Electrophoresis. Test samples were separated using high-pH RP, followed by DDA and DIA analyses using nano-LC–MS/MS. The bioinformatics analysis pipeline was based on sample data generated using a high-resolution mass spectrometer. DDA data were obtained using the Andromeda search engine within MaxQuant, and the results were used for spectral library construction. For large-scale DIA data, the mProphet algorithm was used to complete analytical quality control, thus obtaining a large number of reliable quantitative results. Based on the quantitative outcomes, the ultimately protein sequences are identified from the UniProt database and the protein databases predicated on genome annotation. DIA data were analyzed using iRT peptides for retention time calibration. Based on the target-decoy model applicable to SWATH-MS, a false-positive control was performed with an FDR of 1%, and significant quantitative results were obtained. Msstats [70], an R package from the Bioconductor Repository, was used for the statistical analysis of significant differences in proteins. Screening for differential protein expression was conducted, employing a criterion of a fold change greater than 2 and a P-value less than 0.05 to denote significant differences. Finally, an enrichment analysis was performed on the identified DEPs.

### Time-series analysis

Gene or protein expression profiles in each sugarcane variety during lodging were analyzed using the Short Time-series Expression Miner (STEM) algorithm with default parameters. The DEGs or DEPs in sugarcane were clustered according to their expression trend, and DEGs/DEPs showing different expression trends between two varieties was identified and subjected to KEGG pathway enrichment analysis.

### qRT-PCR analysis

Quantitative reverse transcription PCR (qRT-PCR) was conducted to authenticate the expression of pivotal genes. RNA isolated from the two sugarcane varieties at three stages was transcribed into cDNA utilizing the PrimeScript Reverse Transcriptase kit (Takara). qRT-PCR was executed on the CFX Connect™ Real-Time PCR Detection System (Bio-Rad, Hercules, CA, USA) with specific primers (Table S7). The relative mRNA levels of detected genes were calculated according to the 2^(−ΔCt)^ algorithm and 25S RNA gene was served as an internal control [71].

### Statistical analysis

Three replicates were analyzed for each tissue type at each stage. Pearson correlation coefficients were calculated among the abundances of different genes and proteins from metabolomic profiling and between the relative expression from qRT-PCR and RNA-seq across stages in R v3.6.3.

### Supplementary Information

**Additional file 1: Table S1.** Height data for each period of growth of sugarcane. **Table S2.** The loging resistance index and agronomic trait in different sugarcane varieties (clone) of lodging types. **Table S3.** Contents of biochemical components in different lodging types of  ratoon cane. **Table S4.** Information of the RNA-seq dataset. **Table S5.** Statistics of assembly. **Table S6.** KEGG pathways affected by both gene and protein with differences in trends between varieties. **Table S7.** Primers used in this study.

**Additional file 2: Fig. S1.** Busco assessment of the transcriptomic assembly. **Fig. S2.** The high consistency between transcriptome and qRT-PCR testing.

## Data Availability

The datasets used and/or analysed during the current study are available in the NCBI Bioproject repository, [PRJNA951683].

## Abbreviations

RNA-seq: RNA sequencing
PCA: Principal component analysis
DEGs: Differentially expressed genes
*CV*: Coefficient of variation
DEPs: Differentially expressed proteins
4CL: 4-Coumarat-CoA ligase
CAD: Cinnamyl-alcohol dehydrogenase
SDT1: Pyrimidine and pyridine-specific 5’-nucleotidase
LTI: Lodging tolerance index
NMNAT: Nicotinamide mononucleotide adenylyl transferase
NAD: Nicotinamide adenine dinucleotide
SDT1: Pyrimidine and pyridine-specifc 5’-nucleotidase
NR: Nicotinamide riboside
URH1: Uridine nucleosidase
PPI: Protein–protein interaction
STEM: Short Time Series Expression Miner
ISP: Ionic-soluble pectin
CAP: Covalent-soluble pectin
WSP: Water-soluble pectin

## References

[1] Li Y, Yang L-T (2015). Sugarcane agriculture and sugar industry in China. Sugar Tech.

[2] Xiao Z, Liao X, Guo S (2017). Analysis of sugarcane juice quality indexes. J Food Qual.

[3] Arif S, Batool A, Nazir W, Khan RS, Khalid N. Physiochemical characteristics nutritional properties and health benefits of sugarcane juice. 2019. p. 10.

[4] Yadav R, Solomon S (2006). Potential of developing sugarcane by- product based industries in India. Sugar Tech.

[5] Vaz S (2019). Sugarcane-biorefinery. Adv Biochem Eng Biotechnol.

[6] Manimekalai R, Suresh G, Govinda Kurup H, Athiappan S, Kandalam M (2020). Role of NGS and SNP genotyping methods in sugarcane improvement programs. Crit Rev Biotechnol.

[7] Singh GS, Chapman SC, Jackson PA, Lawn RJ (2002). Lodging reduces sucrose accumulation of sugarcane in the wet and dry tropics. Crop Pasture Sci.

[8] Singh GS, Chapman SC, Jackson PA, Lawn RJ. Lodging - a major constraint to high yield and CCS in the wet and dry tropics. 2000.

[9] Inman-Bamber NG, Bonnett GD, Thorburn PJ, Garside AL, Berding N, Attard S, Bruce RC. Pointers for better farming and research from sugarcane physiology by. 2008.

[10] Ali, Javed. Mechanization of sugarcane cultivation. 2015. 10.13140/RG.2.1.4056.9049.

[11] Dahiya S, Sihag S, Chaudhary C (2018). Lodging: significance and preventive measures for increasing crop production. Int J Chem Stud.

[12] Zhou D, Zhao W, Chen Y, Zhang Q, Deng G, He F (2022). Identification and localisation algorithm for sugarcane stem nodes by combining YOLOv3 and traditional methods of computer vision. Sensors (Basel, Switzerland).

[13] Mulder EG (1954). Effect of mineral nutrition on lodging of cereals. Plant Soil.

[14] Ishimaru K, Togawa E, Ookawa T, Kashiwagi T, Madoka Y, Hirotsu N (2008). New target for rice lodging resistance and its effect in a typhoon. Planta.

[15] Sunday I, Bello B, Abolusoro S, Aremu C (2021). Comparative response of some tropical maize hybrid and their parental varieties to low and high nitrogen regime. Heliyon.

[16] Mizuno H, Kasuga S, Kawahigashi H (2018). Root lodging is a physical stress that changes gene expression from sucrose accumulation to degradation in sorghum. BMC Plant Biol.

[17] Grantz DA (1989). Effect of cool temperatures on photosynthesis and stomatal conductance in field-grown sugarcane in Hawaii. Field Crop Res.

[18] Berry P, Spink J, Sylvester-Bradley R, Pickett A, Sterling M, Baker C, Cameron N. Lodging control through variety choice and management. 2002.

[19] Li H, Guo Y (2012). Mechanical model with varying stiffness and critical grain load of wheat stalk (in Chinese). Nongye Jixie Xuebao/Trans Chin Soc Agric Mach.

[20] Ageeva E, Irina L, Likhenko I (2020). Lodging in wheat: genetic and environmental factors and ways of overcoming. Vavilov J of Genet Breed.

[21] Yang W, Yuan F, Chen YQ, Yang JJ (2020). Effect of root-soil parameters on the lodging resistance of sugarcane (*Saccharum Officinarum L*.). Appl Ecol Environ Res.

[22] Berding N, Hurney AP, Salter B, Bonnett G (2005). Agronomic impact of sucker development in sugarcane under different environmental conditions. Field Crop Res.

[23] Jackson P (2005). Breeding for improved sugar content in sugarcane. Field Crop Res.

[24] Wang L, Liao J, Tan F, Tang S, Huang J, Li X (2015). Breeding of new high-yield, high-sugar and lodging-resistant sugarcane variety Guitang 42 and its high-yield cultivation technique. J Southern Agric.

[25] D’Hont A, Grivet L, Feldmann P, Rao S, Berding N, Glaszmann JC (1996). Characterisation of the double genome structure of modern sugarcane cultivars (*Saccharum spp*.) by molecular cytogenetics. Mol Gen Genet.

[26] Zhang J, Zhang X, Tang H, Zhang Q, Hua X, Ma X (2018). Alleledefined genome of the autopolyploid sugarcane *Saccharum spontaneum L*. Nat Genet.

[27] Olivier G, Gaetan D, Rudie A, Jane G, Bernard P, Karen A (2018). A mosaic monoploid reference sequence for the highly complex genome of sugarcane. Nat Commun.

[28] Li AM, Wang M, Chen ZL, Qin CX, Liao F, Wu Z, He WZ, Lakshmanan P, Pan YQ, Huang DL (2022). Integrated transcriptome and metabolome analysis to identify sugarcane gene defense against fall armyworm (*Spodoptera frugiperda*) herbivory. Int J Mol Sci.

[29] Manimekalai R, Suresh G, Singaravelu B. Sugarcane transcriptomics in response to abiotic and biotic stresses: a review. Sugar Tech. 2022;24. 10.1007/s12355-021-01098-9.

[30] tamp, P. & Kiel, C.  (2008). Root morphology of maize and its relationship to root lodging. J Agron Crop Sci.

[31] Nakajima T, Yoshida M, Tomimura K (2008). Effect of lodging on the level of mycotoxins in wheat, barley, and rice infected with the *Fusarium graminearum* species complex. J Gen Plant Pathol.

[32] Wang D, Ding WH, Feng SW, Hu TZ, Li G, Li XH, Yang YY, Ru ZG (2016). Stem characteristics of different wheat varieties and its relationship with lodging-resistance. J Appl Ecol.

[33] Ma QH (2009). The expression of caffeic acid 3-O-methyltransferase in two wheat genotypes differing in lodging resistance. J Exp Bot.

[34] Terrett OM, Dupree P (2019). Covalent interactions between lignin and hemicelluloses in plant secondary cell walls. Curr Opin Biotechnol.

[35] Li Q, Fu C, Liang C, Ni X, Zhao X, Chen M, Ou L (2022). Crop lodging and the roles of lignin, cellulose, and hemicellulose in lodging resistance. Agronomy.

[36] Zhang J, Li G, Song Y, Liu Z, Yang C, Tang S, Zheng C, Wang S, Ding Y (2014). Lodging resistance characteristics of high-yielding rice populations. Field Crop Res.

[37] Kumar M, Campbell L, Turner SR (2016). Secondary cell walls: biosynthesis and manipulation. J Exp Bot.

[38] Kong E, Liu D, Guo X, Yang W, Sun J, Li X, Zhan K, Cui D, Lin J, Zhang A (2013). Anatomical and chemical characteristics associated with lodging resistance in wheat. Crop J.

[39] Ming X (2000). Study on conducting bundle character of reck and correlation of several rice breeds. J Agric Sci Yanbian Univ..

[40] Shah L, Yahya M, Shah SMA, Nadeem M, Ali A, Ali A, Wang J, Riaz MW, Rehman S, Wu W, Khan RM, Abbas A, Riaz A, Anis GB, Si H, Jiang H, Ma C (2019). Improving lodging resistance: using wheat and rice as classical examples. Int J Mol Sci.

[41] Boerjan W, Ralph J, Baucher M (2003). Lignin biosynthesis. Annu Rev Plant Biol.

[42] Vogt T (2010). Phenylpropanoid biosynthesis. Mol Plant.

[43] Liu L, Liu S, Lu H, Tian Z, Zhao H, Wei D, Wang S, Huang Z (2022). Integration of transcriptome and metabolome analyses reveals key lodging-resistance-related genes and metabolic pathways in maize. Front Genet.

[44] Yoon J, Choi H, An G (2015). Roles of lignin biosynthesis and regulatory genes in plant development. J Integr Plant Biol.

[45] Mutwil M, Debolt S, Persson S (2008). Cellulose synthesis: a complex complex. Curr Opin Plant Biol.

[46] Li Y, Qian Q, Zhou Y, Yan M, Sun L, Zhang Mu, Fu Z, Wang Y, Han B, Pang X, Chen M, Li J (2003). BRITTLE CULM1, which encodes a COBRA-like protein, affects the mechanical properties of rice plants. Plant Cell.

[47] Yang S, Chu N, Feng N, Zhou B, Zhou H, Deng Z, Shen X, Zheng D (2023). Global responses of autopolyploid sugarcane badila (Saccharum officinarum L.) to drought stress based on comparative transcriptome and metabolome profiling. Int J Mol Sci.

[48] Dong NQ, Lin HX (2021). Contribution of phenylpropanoid metabolism to plant development and plant-environment interactions. J Integr Plant Biol.

[49] Abadie C, Tcherkez G (2019). Plant sulphur metabolism is stimulated by photorespiration. Commun Biol.

[50] Zheng M, Chen J, Shi Y, Li Y, Yin Y, Yang D (2016). Manipulation of lignin metabolism by plant densities and its relationship with lodging resistance in wheat. Sci Rep.

[51] Ma QH, Luo HR (2015). Biochemical characterization of caffeoyl coenzyme A 3-O-methyltransferase from wheat. Planta.

[52] Yan B, Zhang Z, Zhang P, Zhu X, Jing Y, Wei J (2019). Nitric oxide enhances resistance against black spot disease in muskmelon and the possible mechanisms involved. Sci Hortic.

[53] Gui J, Shen J, Li L (2011). Functional characterization of evolutionarily divergent 4-coumarate: coenzyme a ligase in rice. Plant Physiol.

[54] Dutilleul C, Driscoll SP, Cornic G, de Paepe R, Foyer CH, Noctor G (2003). Functional mitochondrial complex I Is required by tobacco leaves for optimal photosynthetic performance in photorespiratory conditions and during transients1. Plant Physiol.

[55] Dutilleul C, Garmier M, Noctor G, Mathieu C, Chétrit P, Foyer CH, de Paepe R (2003). Leaf Mitochondria modulate whole cell redox homeostasis, set antioxidant capacity, and determine stress resistance through altered signaling and diurnal regulation article, publication date, and citation information can be found at www.plantcell.org/cgi/doi/10.1105/tpc.009464. Plant Cell Online.

[56] Chen W, Wang W, Peng M, Gong L, Gao Y, Wan J, Wang S, Shi L, Zhou B, Li Z, Peng X, Yang C, Qu L, Liu X, Luo J (2016). Comparative and parallel genome-wide association studies for metabolic and agronomic traits in cereals. Nat Commun..

[57] Lau C, Niere M, Ziegler M (2009). The NMN/NaMN adenylyltransferase (NMNAT) protein family. Front Biosci (Landmark edition).

[58] Bogan KL, Evans C, Belenky P, Song P, Burant CF, Kennedy R, Brenner C (2009). Identification of *Isn1* and *Sdt1* as glucose- and vitamin-regulated nicotinamide mononucleotide and nicotinic acid mononucleotide [corrected] 5'-nucleotidases responsible for production of nicotinamide riboside and nicotinic acid riboside. J Biol Chem.

[59] De Sousa Abreu R, Penalva LO, Marcotte EM, Vogel C (2009). Global signatures of protein and mRNA expression levels. Mol. Biosyst..

[60] Kumar Dhirendra, Bansal Gourja, Narang Ankita, Basak Trayambak, Abbas Tahseen, Dash Debasis (2016). Integrating transcriptome and proteome profiling: Strategies and applications. Proteomics..

[61] Liu C, Liu Y, Guo K, Wang S, Yang Y (2014). Concentrations and resorption patterns of 13 nutrients in different plant functional types in the karst region of south-western China. Ann Bot.

[62] Neumann M, Nöske R, Bach G, Glaubauf T, Bartoszek M, Strauch P (2011). A procedure for rapid determination of the silicon content in plant materials. Zeitschrift für Naturforschung B.

[63] Banerjee P, Prasad B (2020). Determination of concentration of total sodium and potassium in surface and ground water using a flame photometer. Appl Water Sci.

[64] Javier-Astete R, Jimenez-Davalos J, Zolla G (2021). Determination of hemicellulose, cellulose, holocellulose and lignin content using FTIR in Calycophyllum spruceanum (Benth.) K Schum. and Guazuma crinita Lam. PloS One..

[65] Wenlu Hao, Hengxin Liu, Liang Zhu (2008). Measuring Methods for Agricultural Machinery Testing Conditions-General Rules.

[66] Li X. Evaluation of lodging resistance in sugarcane (*Saccharum spp. hybrid*) germplasm resources. Appl Ecol Environ Res. 2019;3:6107–16.

[67] Shao QQ, Zhou Q, Wang X, Cai J, Huang M, Dai TB, Jiang D (2018). Morphological and anatomical characteristics of wheat varieties and its response to paclobutrazol. J Triticeae Crops.

[68] Soffan A, Subandiyah S, Wijonarko A, Sawitri DW (2021). RNA-seq data of tea mosquito bugs, *Helopeltis bradyi*, antennae. Data Brief.

[69] Love MI, Huber W, Anders S (2014). Moderated estimation of fold change and dispersion for RNA-seq data with DESeq2. Genome Biol.

[70] Choi Meena, Chang Ching-Yun, Clough Timothy, Broudy Daniel, Killeen Trevor, MacLean Brendan, Vitek Olga (2014). MSstats: an R package for statistical analysis of quantitative mass spectrometry-based proteomic experiments. Bioinformatics (Oxford, England)..

[71] Winer J, Jung CK, Shackel I, Williams PM (1999). Development and validation of real-time quantitative reverse transcriptase-polymerase chain reaction for monitoring gene expression in cardiac myocytes in vitro. Anal Biochem.

