# Supplementary material for: Integrated transcriptomic and proteomic analyses of two sugarcane (Saccharum officinarum Linn.) varieties differing in their lodging tolerance
Source: BMC Plant Biol. 2023 Nov 29;23:601. doi: 10.1186/s12870-023-04622-z (PMC10685470; doi:10.1186/s12870-023-04622-z)
Supplement: Supplementary file 2 — Additional file 2: Fig. S1. Busco assessment of the transcriptomic assembly. Fig. S2. The high consistency between transcriptome and qRT-PCR testing. [file 12870_2023_4622_MOESM2_ESM.pdf]

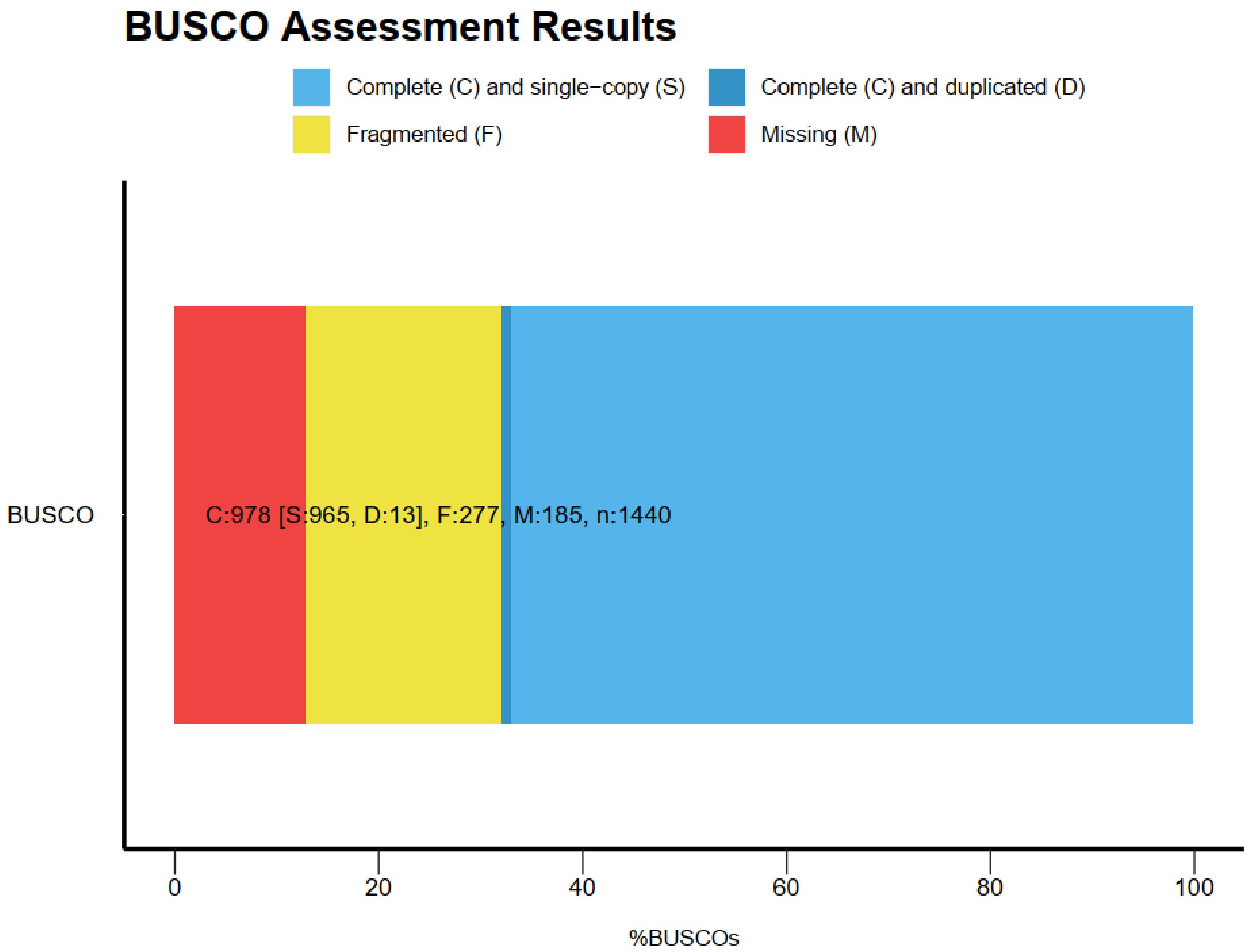

Fig. S1 Busco assessment of the transcriptomic assembly.

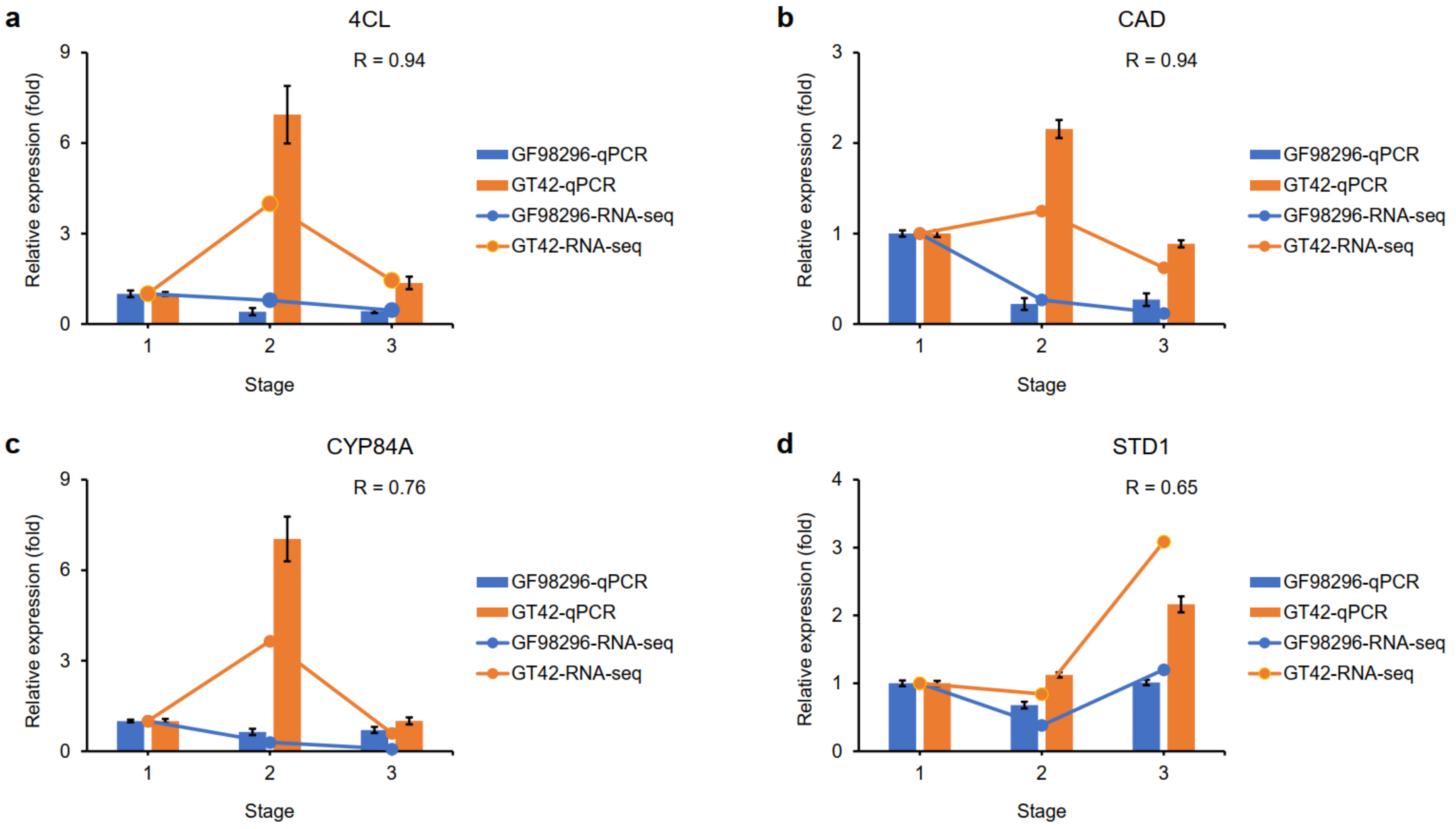

Fig.S2. The high consistency between transcriptome and qRT-PCR testing
